# Anti-podocin Enzyme-Linked Immunosorbent Assay Guides Immunotherapy in Steroid-Resistant Nephrotic Syndrome

**DOI:** 10.1016/j.ekir.2025.07.003

**Published:** 2025-07-16

**Authors:** Valentina Raglianti, Luigi Cirillo, Maria Lucia Angelotti, Letizia De Chiara, Benedetta Mazzinghi, Giulia Antonelli, Carolina Conte, Maria Elena Melica, Anna Julie Peired, Elena Lazzeri, Laura Lasagni, Viviana Palazzo, Samuela Landini, Anna Maria Buccoliero, Samantha Innocenti, Carmela Errichiello, Elisa Buti, Giulia Sansavini, Andrea La Tessa, Francesca Becherucci, Hans-Joachim Anders, Paola Romagnani

**Affiliations:** 1 Nephrology and Dialysis Unit, Meyer Children’s Hospital IRCCS, Florence, Italy; 2 Department of Biomedical, Experimental and Clinical Sciences “Mario Serio,” University of Florence, Florence, Italy; 3 Pathology Unit, Meyer Children’s Hospital IRCCS, Florence, Italy; 4 Division of Nephrology, Department of Medicine IV, Hospital of Ludwig Maximilians University Munich, Munich, Germany

## Introduction

Steroid-resistant nephrotic syndrome (SRNS) has diverse causes; however, treatment often relies on high-dose corticosteroids, calcineurin inhibitors, mycophenolate mofetil, cyclophosphamide, or rituximab (RTX) in a trial-and-error fashion, without identifying the underlying etiology.^1^ Recently, autoantibodies against slit diaphragm proteins—nephrin, podocin, and Kirrel1—have been found in patients responsive to second-line immunosuppression, but not in genetic SRNS.^2^, ^3^, ^4^, ^5^, ^6^ However, diagnostic tools for detecting autoimmune podocytopathies (AuPs) remain limited. We and others recently developed enzyme-linked immunosorbent assays (ELISA) to detect anti-nephrin (AN), anti-podocin (AP), and anti-Kirrel1 (AK) IgG, enabling diagnosis of ANAuP, APAuP, and AKAuP as distinct autoimmune causes of SRNS.^2^, ^3^, ^4^, ^5^, ^6^

Here, we report a case where the novel AP IgG ELISA could: (i) clarify autoimmunity as a cause of SRNS, (ii) identify lack of immunological response to steroids underlying the incomplete clinical response, and c) identify immediate relapse after an initial immunological response to RTX. In addition, monitoring serum AP IgG levels was key in guiding the decision for deep B-cell depletion with obinutuzumab and in confirming the immunological response to the latter.

## Case Presentation

**Figure 1:**
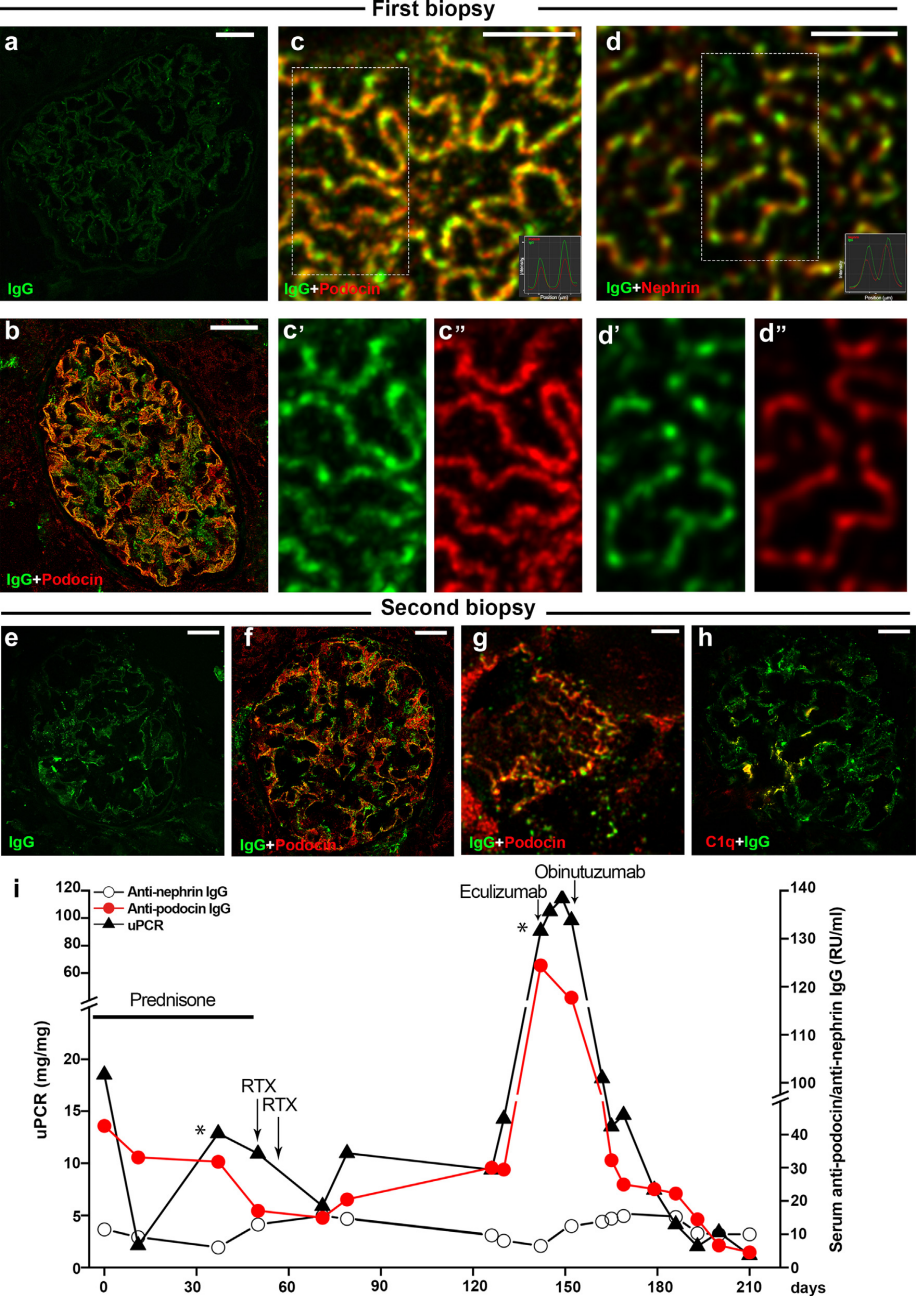
Integrated assessment of clinical progression, autoimmune profile, and renal biopsy results. In the first kidney biopsy: (a) representative image of routine immunofluorescence revealing the absence of glomerular deposition of IgG. Bar = 25 μm. (b) Representative image of high-resolution confocal microscopy detecting IgG (green) deposition along the slit diaphragm, colocalized (yellow) with podocin (red). Bar = 25 μm. (c) Representative super resolution STED microscopy image confirming IgG (green) colocalization (yellow) with podocin (red) and the details split by channel (C’, C’’) Bar = 2 μm. Representative fluorescence intensity profile plot (inlet) showing the complete overlap between IgG (green) and podocin (red) signals. (d) Representative super resolution STED microscopy showing no IgG (green) colocalization with nephrin (red) and the details split by channel (D’, D’’). Bar = 2 μm. Representative fluorescence intensity profile plot (inlet) showing the lack of overlap between IgG (green) and nephrin (red) signals. In the second biopsy: (e) representative image of routine immunofluorescence showing a mild deposition of IgG. (f) Representative image of high-resolution confocal microscopy showing that a part of IgG (green) colocalized (yellow) with podocin (red). Bar = 25 μm. A detail in (g). (h) Representative image of immunofluorescence staining showing colocalization (yellow) of IgG (green) and C1q (red). Bar = 25 μm. (i) Time course showing the consistently aligned trends of proteinuria (black triangle) and serum anti-podocin IgG levels (red dot), which move in line. In contrast, serum antinephrin IgG levels (empty dot) do not follow the same pattern. Values are expressed in relative units (RU/ml) due to the patient’s severe persistent hypogammaglobulinemia. RTX, rituximab; STED, stimulated emission depletion; uPCR, urinary protein-to-creatinine ratio. ∗kidney biopsy.

A 5-year-old White female presented with sudden onset NS shortly after an upper respiratory tract infection. Initial laboratory evaluation showed an increased urinary protein-to-creatinine ratio of 18.5 mg/mg, reduced levels of serum albumin (1.39 g/dl) and IgG (139 mg/dl), but an elevated low-density lipoprotein cholesterol of 295 mg/dl. The serum creatinine of 0.39 mg/dl was normal for her age and body mass. After clinical exclusion of secondary causes, treatment with prednisone 60 mg/m^2^/d was initiated. Concurrently, the patient developed symptoms and laboratory findings consistent with *de novo* type 1 diabetes mellitus controlled well with insulin. Over 4 weeks, proteinuria did not change, so the patient was classified as SRNS. Antiproteinuric therapy with ramipril was initiated and a kidney biopsy was performed (Figure 1a–d). Routine light microscopy revealed minimal changes (Supplementary Figure S1A) and standard immunofluorescence was negative (Figure 1a). However, high-resolution microscopy localized IgG deposits along the filtration slit (Figure 1b). Super-resolution stimulated emission depletion microscopy further revealed that IgG colocalized with podocin and not with nephrin (Figure 1c–d). Subsequent serum ELISA testing identified the presence of AP IgG, while AN and AK IgG were absent, supporting the diagnosis of a steroid-resistant APAuP and excluding ANAuP or AKAuP (Table 1 and Supplementary Figure S1B). Whole-exome sequencing excluded a monogenic podocytopathy or phenocopies.

**Table 1:** Teaching points

Teaching point	Explanation
1. Anti-podocin ELISA enables rapid diagnosis of APAuP	Identifies AuP and distinguishes it from genetic SRNS or other autoimmune forms (e.g., ANAuP, AKAuP).
2. Biomarker tracking guides clinical decisions	Serial anti-podocin IgG levels reflect disease activity, supporting real-time monitoring.
3. ELISA-based diagnosis avoids non-targeted immunosuppression	Enabled a rational choice of obinutuzumab, avoiding conventional agents like CNIs, MMF, or CF.
4. Obinutuzumab is effective in RTX-resistant APAuP	Deep B-cell depletion induced clinical and immunological remission where RTX had failed.
5. AuP IgG can associate with complement-mediated RPGN	Rising antibody levels during infection correlated with RPGN, hematuria, and C1q/IgG colocalization in renal tissue.
7. APAuP may coexist with type 1 diabetes	Suggests possible shared autoimmune mechanisms or cross-reactivity, as podocin is expressed in pancreatic tissue.
8. Repeat kidney biopsy may be necessary in complex SRNS cases	Helped identify active immune pathology and complement deposition, guiding escalation to targeted therapy.

AKAuP, anti-Kirrel1 AuP; ANAuP, antinephrin AuP; APAuP, antipodocin AuP; AuP, autoimmune podocytopathy; CF, cyclophosphamide; CNI, calcineurin inhibitors; ELISA, enzyme-linked immunoadsorbent assay; MMF, mycophenolate mofetil; RPGN, rapidly progressive glomerulonephritis; RTX, rituximab; SRNS, steroid-resistant nephrotic syndrome.

After having confirmed the diagnosis of an AuP underlying the severe SRNS (Figure 1c–d) and Supplementary Figure S1A and B), 2 doses of RTX (375 mg/m^2^) were administered 2 weeks apart (Figure 1i) followed by complete depletion of circulating CD19+ B cells (Supplementary Figure S1F). RTX therapy resulted in a decline of urinary protein-to-creatinine ratio from 11 to 5.9 mg/mg (Figure 1i) and edema, hypoalbuminemia, as well as hypogammaglobulinemia improved.

Two weeks after the second RTX dose, the patient developed a respiratory infection with a throat swab positive for *Chlamydia pneumoniae*. Thoracic ultrasound and x-rays confirmed bilateral pneumonia, thus antibiotic treatment with macrolides was started. The infection triggered a sudden relapse of severe NS with diuretic-resistant edema and a corresponding increase in the urinary protein-to-creatinine ratio (11–98 mg/mg), new onset of microhematuria and leukocyturia, and serum creatinine increase from 0.33 mg/dl to 1.3 mg/dl (Figure 1i and Supplementary Figure S1F). This relapse of NS was accompanied with serum AP IgG levels increasing up to 3 times the levels detected at onset, whereas AN IgG levels remained negative (Figure 1i and Table 1). In Addition, functional complement assays indicated classical complement pathway activation (Wieslab test, increased sC5b-9, increased c3d).

With the clinical suspect of an infection-triggered rapidly progressive glomerulonephritis (RPGN) superimposed on SRNS, we performed a second kidney biopsy which confirmed a proliferative GN with few crescents, as well as IgG and C3 deposition along the glomerular basement membrane (Figure 1e, Table 1, and Supplementary Figure S1C and D). High-resolution microscopy revealed extensive and severe disruption of the slit diaphragm, with a granular pattern of IgG and podocin colocalization (Figure 1f and g). Furthermore, a part of IgG deposits showed colocalization with C1q, confirming activation of the classical complement pathway (Figure 1h). Given the RPGN, evidence of classical complement pathway activation, and aiming to avoid further corticosteroid use for the concomitant type 1 diabetes mellitus, we used the complement C5 blocker eculizumab (Figure 1i and Supplementary Figure S1F). This therapy induced a rapid decline in serum creatinine from 1.3 mg/dl to 0.7 mg/dl, but no improvement in NS, suggesting a good treatment effect of eculizumab on the infection-related RPGN (Figure 1i and Supplementary Figure S1F).

However, serum creatinine did not decrease further below 0.7 mg/dl, and severe NS persisted along with high serum levels of AP IgG (Figure 1i and Supplementary Figure S1F). In this constellation of RTX-resistant APAuP, we did not consider readministering eculizumab or using a calcineurin inhibitor, mycophenolate mofetil, or cyclophosphamide (Table 1). Instead, we opted to treat this proven form of an autoimmune NS like other RTX-resistant autoimmune diseases with the second-generation B-cell–depleting drug, obinutuzumab (Figure 1i, Table 1, and Supplementary Figure S1F). Upon treatment with 1 g/m^2^ obinutuzumab, serum AP IgG levels rapidly declined as did serum creatinine levels to 0.3 mg/dl. This was followed by a gradual improvement in proteinuria up to urinary protein-to-creatinine ratio of 1 mg/mg and a complete clinical remission of NS (Figure 1i and Supplementary Figure S1F).

## Discussion

This case illustrates the complexity of RTX-resistant SRNS in a pediatric patient, complicated by RPGN triggered by pneumonia. This case underscores 3 key points as follows:

(i) Diagnostic utility of AP ELISA: serum AP IgG provided a rapid, noninvasive diagnosis of AuP (i.e., APAuP), distinguishing it from other forms such as ANAuP and AKAuP. In our case, only AP IgG was detected, whereas AN and AK antibodies were absent. Given the technical challenges associated with AN ELISA,^4^ all 3 assays were previously validated in a large idiopathic NS cohort by comparing serum results with IgG presence and colocalization with nephrin, podocin, or Kirrel1 in kidney biopsies of the same patients, assessed by using stimulated emission depletion microscopy.^5^^,^^6^ Notably, the AP ELISA showed high diagnostic accuracy, with 100% sensitivity and 97% specificity, as previously detailed.^6^ This ELISA works similarly to anti-phospholipase A2 receptor antibody testing and allows repeated measurement to guide treatment.^7^
(ii). Monitoring disease activity: beyond diagnosis, AP IgG ELISA enabled real-time monitoring of disease activity, and outperformed CD19-based B-cell tracking. In APAuP, like ANAuP,^4^ proteinuria improved in parallel with antibody decline; unlike phospholipase A2 receptor antibody–related disease, where immune deposits delay remission.^7^ This correlation helps explain varying clinico-pathological presentations in antibody-mediated podocytopathies.
(iii). Therapeutic guidance: the identification of AP antibodies directly influenced treatment, avoiding a trial-and-error approach. Conventional second-line therapies (calcineurin inhibitor, mycophenolate mofetil, and cyclophosphamide) were bypassed, because the ELISA pointed to autoreactive B cells as the pathogenic driver. Obinutuzumab was selected for its superior B-cell depleting efficacy, supported by experience in RTX-resistant phospholipase A2 receptor antibody–positive NS and lupus nephritis.^8^

This case also offers broader clinical insights. First, APAuP may coexist with type 1 diabetes. This overlap could reflect shared autoimmune predisposition or cross-reactivity, because podocin is expressed in pancreatic tissue.^9^ Second, APAuP may evolve into RPGN with hematuria and complement activation, particularly in the setting of *Chlamydia pneumoniae* infection. In this case, eculizumab led to transient improvement; however, NS persisted until AP IgG was cleared, suggesting that extremely high AP IgG levels—amplified during pneumonia—may have contributed to this severe course. Indeed, AP antibodies levels mirrored not only NS progression, but also renal function decline and hematuria onset.

In summary, serum ELISA for anti–slit diaphragm antibodies offers a promising tool for diagnosing and guiding treatment of AuPs.

## Disclosure

H-JA received consulting fees from AstraZeneca, Novartis, GSK, Janssen, Bayer, Boehringer-Ingelheim, Roche, Lilly, and Otsuka; research funding from ERA-Per-Med grant from the BMBF, Deutsche Forschungsgemeinschaft AN372/30-1; and has a leadership or fiduciary role with ERA EiC of NDT. PR, FB, LC, VR, SL, and VP are members of the ERKNet. All the other authors declared no competing interests. The ELISA assay described in this article is subject of a patent (PCT/IB2024/060751).

## Patient Consent

Ethical approval (number 224/2021) and parental consent were obtained for the publication of this case report.

### Funding

This project has received funding from the 10.13039/501100007601European Union’s Horizon 2020 research and innovation programme and from Tuscany Region under the ERA-Net Cofund in Personalised Medicine ERA PerMed (G.A. No. 779282) to PR, EL, H-JA (01KU2204). The findings and conclusions in this report are those of the authors and do not necessarily represent the official position of the funding institution.

## Supplementary Material

Supplementary File (PDF) High resolution confocal microscopy. Stimulated emission depletion microscopy. Enzyme-Linked Immunosorbent Assay (ELISA) for anti-podocin autoantibodies. **Figure S1.** Histological findings and longitudinal assessment of antibody titers, urinary parameters, and renal function in response to treatments.

## Footnotes

Supplementary File (PDF)
High resolution confocal microscopy. Stimulated emission depletion microscopy. Enzyme-Linked Immunosorbent Assay (ELISA) for anti-podocin autoantibodies. **Figure S1.** Histological findings and longitudinal assessment of antibody titers, urinary parameters, and renal function in response to treatments.

## References

[1] Kopp J.B., Anders H.J., Susztak K. (2020). Podocytopathies. Nat Rev Dis Primers.

[2] Watts A.J.B., Keller K.H., Lerner G. (2022). Discovery of autoantibodies targeting Nephrin in minimal change disease supports a novel autoimmune etiology. J Am Soc Nephrol.

[3] Shirai Y., Miura K., Ishizuka K. (2024). A multi-institutional study found a possible role of anti-nephrin antibodies in post-transplant focal segmental glomerulosclerosis recurrence. Kidney Int.

[4] Hengel F.E., Dehde S., Lassé M. (2024). Autoantibodies targeting Nephrin in podocytopathies. N Engl J Med.

[5] Raglianti V., Angelotti M.L., Cirillo L. (2024). Anti-slit diaphragm antibodies on kidney biopsy identify pediatric patients with steroid-resistant nephrotic syndrome responsive to second-line immunosuppressants. Kidney Int.

[6] Raglianti V., Angelotti M.L., De Chiara L. (2025). Anti-slit antibodies against podocin and Kirrel1 in pediatric and adult podocytopathies. J Am Soc Nephrol.

[7] Ronco P., Beck L., Debiec H. (2021). Membranous nephropathy. Nat Rev Dis Primers.

[8] Rossi G.M., Baier E., Vaglio A. (2025). Obinutuzumab for the management of immune-mediated glomerular diseases. Nephrol Dial Transplant.

[9] Rinta-Valkama J., Palmén T., Lassila M., Holthöfer H. (2007). Podocyte-associated proteins FAT, alpha-actinin-4 and filtrin are expressed in Langerhans islets of the pancreas. Mol Cell Biochem.

