# Supplementary material for: Anti-podocin Enzyme-Linked Immunosorbent Assay Guides Immunotherapy in Steroid-Resistant Nephrotic Syndrome
Source: Kidney Int Rep. 2025 Jul 16;10(10):3689–93. doi: 10.1016/j.ekir.2025.07.003 (PMC12545812; doi:10.1016/j.ekir.2025.07.003)
Supplement: Supplementary File (PDF) — High resolution confocal microscopy. Stimulated emission depletion microscopy. Enzyme-Linked Immunosorbent Assay (ELISA) for anti-podocin autoantibodies. Figure S1. Histological findings and longitudinal assessment of antibody titers, urinary parameters, and renal function in response to treatments. [file mmc1.pdf]

SUPPLEMENTARY MATERIAL

Anti-podocin ELISA guides immunotherapy in steroid-resistant nephrotic syndrome

Valentina Raglianti[1,2], Luigi Cirillo[1,2], Maria Lucia Angelotti[2], Letizia De Chiara[2], Benedetta Mazzinghi[1], Giulia Antonelli[2], Carolina Conte[2], Maria Elena Melica[2], Anna Julie Peired[2], Elena Lazzeri[2], Laura Lasagni[2], Viviana Palazzo[1], Samuela Landini[1], Anna Maria Buccoliero[3], Samantha Innocenti[1], Carmela Errichiello[1], Elisa Buti[1], Giulia Sansavini[1], Andrea La Tessa[1], Francesca Becherucci[1,2], Hans-Joachim Anders[4], Paola Romagnani[1,2].

[*]Valentina Raglianti, Luigi Cirillo and Maria Lucia Angelotti contributed equally to this work

Supplementary material

High resolution confocal microscopy 1

STED microscopy 1

Enzyme-Linked Immunosorbent Assay (ELISA) for anti-podocin autoantibodies 2

Supplementary figures

Figure S1. 3

High resolution confocal microscopy

For confocal microscopy, 10 µm cryosections were stained, as previously reported.[2] Immunolabeling experiments were done in a blinded fashion by two independent investigators. Briefly, sections were fixed in 95% cold ethanol for 10 minutes and subsequently blocked for one hour at room temperature with phosphate buffer saline supplemented with 2% bovine serum albumin and 2% fetal bovine serum. All antibodies were diluted in this blocking solution and incubated for one hour at room temperature. Podocin was detected using 20 µg/ml primary polyclonal rabbit anti-human podocin (Merck; P0372), followed by a secondary AlexaFluor 555-conjugated donkey anti-rabbit IgG (Invitrogen; A-31572) diluted 1:500. IgG immune deposits were detected using a primary monoclonal mouse anti-human IgG antibody (Abcam; ab200699) diluted 1:750, followed by a secondary AlexaFluor 488-conjugated goat anti-mouse IgG2a (Invitrogen; A-21131) diluted 1:500. Images were acquired on a Leica Stellaris 5 confocal microscope, with Leica HC PL APO CS2 63×/1.40 oil objective and highly sensitive and low-noise hybrid detectors (HyD). In order to obtain a high resolution (i.e. 140 nm), we used sub-airy unit pinhole size (0.5 AU) and deconvolution algorithms (Huygens Professional software 24.04 version, Scientific Volume Imaging B.V.) to benefit from a superior performance of the confocal device.

STED microscopy

For STED microscopy, 10 µm cryosections were fixed in 95% cold ethanol for 10 minutes and subsequently blocked for one hour at room temperature with phosphate buffer saline supplemented with 2% bovine serum albumin and 2% fetal bovine serum. All antibodies were diluted in this blocking solution and incubated for one hour at room temperature. Nephrin was detected using 4 µg/ml primary polyclonal sheep anti-human nephrin (R&D Systems; AF4269), followed by a secondary AlexaFluor 555-conjugated donkey anti-sheep IgG (Invitrogen; A-21436) diluted 1:100. Podocin was detected using 20 µg/mL primary polyclonal rabbit anti podocin (Merck, P0372), followed by a secondary antibody AlexaFluor 555 donkey anti-rabbit IgG (Invitrogen; A-31572) diluted 1:100. Kirrel1 was detected using a primary polyclonal rabbit anti Kirrel1 (Merck, ABS1211) diluted 1:50, followed by a secondary antibody AlexaFluor 555 donkey anti-rabbit IgG (Invitrogen; A-31572) diluted 1:100. IgG immune deposits were detected using a primary monoclonal mouse anti-human IgG antibody (Abcam; ab200699) diluted 1:250, followed by a secondary AlexaFluor 532-conjugated goat anti-mouse IgG (Invitrogen; A-11002) diluted 1:100. Immunolabeling experiments were done in a blinded fashion by two independent investigators. Images were acquired by using an SP8 STED 3X confocal microscope (Leica Microsystems) and deconvolved with Huygens

Professional software. A 660 nm pulsed depletion laser was used with a gating between 0.5 and 6 ns. Images were acquired with Leica HC PL APO 93X/1.3 GLYC motCORR STED white objective.

Enzyme-Linked Immunosorbent Assay (ELISA) for anti-Podocin and anti-Kirrel1 autoantibodies

Circulating anti-Podocin antibodies were quantified in the serum of patients using ELISA assay performed according to the following protocol, as previously described[6]. ELISA plates were coated with 100 ng/well of recombinant human podocin (PO 9287 R&D, Biotechne) and incubated O/N at 4°C. Uncoated control wells were used to determine nonspecific binding (in the absence of antigen) for each patient sample, enabling background subtraction. The plates were washed three times with PBS containing 0.05% Tween 20 (PBST) and then were blocked with SuperBlock (Thermo Fisher). Plates were incubated with patient samples. Plates were washed five times with PBST followed by incubation with biotin-conjugated goat anti-human IgG Fc, highly cross-absorbed antibody (Thermo Fisher) diluted to 0.75 µg/ml in SuperBlock containing 0.1% Tween 20 (SuperT). Plates were washed five times with PBST followed by incubation with of HRP-conjugated avidin diluted to 1:2000 in SuperT. Following five final washes with PBST, TMB substrate (BioLegend) and then stop solution (BioLegend) were added. Absorbance was measured at 450 nm. Background correction was performed by subtracting the average OD of uncoated wells from that of antigen-coated wells for each patient sample.

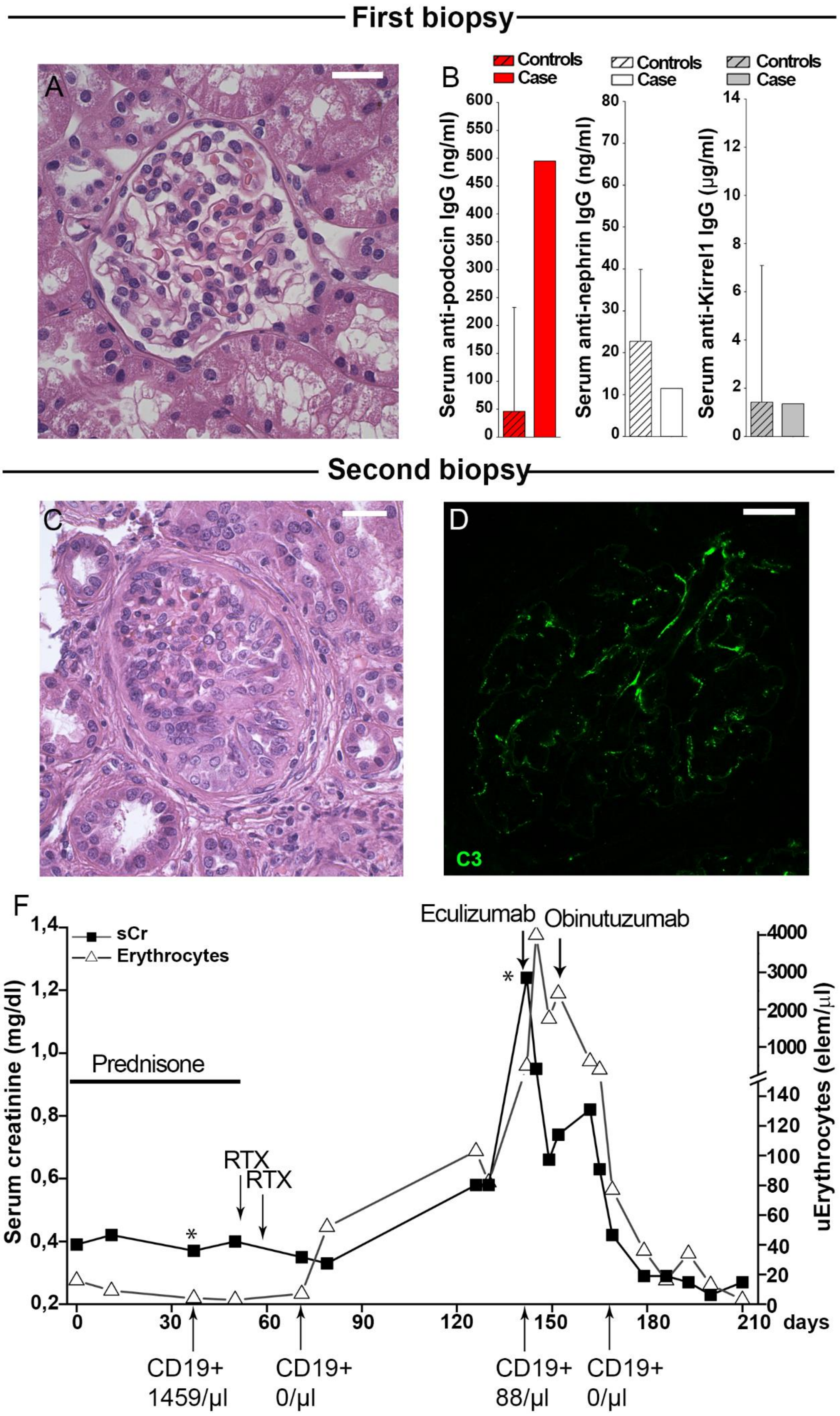
First biopsy
A
B
Controls
Case
Controls
Case
Controls
Case
Serum anti-podocin IgG (ng/ml)
Serum anti-nephrin IgG (ng/ml)
Serum anti-Kirrel1 IgG (µg/ml)
Second biopsy
C
D
C3
F
sCr
Erythrocytes
Serum creatinine (mg/dl)
uErythrocytes (elem/µl)
Prednisone
RTX
RTX
Eculizumab
Obinutuzumab
days
CD19+ 1459/µl
CD19+ 0/µl
CD19+ 88/µl
CD19+ 0/µl

Figure S1. Histological findings and longitudinal assessment of antibody titers, urinary parameters, and renal function in response to treatments. (A) Light microscopy representative image showing a glomerulus without significant histologic changes. Bar = 25 μm. (B) Serum IgG levels by ELISA testing at onset showing the presence of anti-podocin IgG and the absence of anti-nephrin and anti-Kirrel1 IgG. (C) Light microscopy representative image showing a glomerulus with lobular architecture, glomerular hypercellularity with mesangial expansion and infiltration of inflammatory cells. Bar = 25 μm. In the second biopsy: (D) Representative image of routine immunofluorescence showing a mild deposition of C3. (F) Time course of changes in serum creatinine (black square) and urinary erythrocytes (empty triangle). RTX = rituximab; sCr = serum creatinine**;** * kidney biopsy.
